# Psychosocial stress, demoralization and the consumption of tobacco, alcohol and medical drugs by veterinarians

**DOI:** 10.1186/1745-6673-4-4

**Published:** 2009-02-25

**Authors:** Melanie Harling, Petra Strehmel, Anja Schablon, Albert Nienhaus

**Affiliations:** 1 Institution for Statutory Accident Insurance and Prevention in the Health and Welfare Services, Department of Occupational Health Research, Pappelallee 35-37, 22089 Hamburg, Germany; 2 Faculty of Business and Social Work, Hamburg University of Applied Sciences, Saarlandstrasse 30, 22303 Hamburg, Germany

## Abstract

**Background:**

In this cross-sectional study the association between psychosocial stress, demoralization and the consumption of psychotropic substances in veterinarians was examined using data from a sample of 1,060 subjects (52.7% response).

**Methods:**

Multiple logistic regression models were used to determine risk factors for psychosocial stress, demoralization, tobacco consumption (≹ 10 items/day), high-risk alcohol consumption (men > 20 g pure alcohol/day, women > 10 g pure alcohol/day), binge drinking, problem drinking according to CAGE and regular medical drug intake (at least weekly).

**Results:**

Intense psychosocial stress is a risk factor for binge drinking and for regular drug use. High demoralization values are associated with tobacco consumption, problem drinking and regular drug intake. The probability of a high demoralization value increased with intense psychosocial stress.

Practicing veterinarians are more frequently affected by psychosocial stress and have a greater risk of alcohol or drug consumption than veterinarians working in a non-clinical area of work (e.g. Department of Veterinary Services, Industry).

**Conclusion:**

The findings support the hypothesis of complex interrelationships between psychosocial stress, demoralization and the consumption of psychotropic substances in the veterinary profession and underscore the need of further research.

## Background

Veterinarians are exposed to a variety of risks at work. These include injuries by the animals being treated, traffic and travel accidents and diseases of the skin and respiratory tract [1-3]. Moreover, a study from New Zealand [4] and one longitudinal Australian study [5] report a considerable amount of stress in the veterinary profession. In Germany very little is known about this topic. Only one study by Trimpop et al. [6] on accident rates in veterinary surgeries also examined job-related stress and job satisfaction.

Furthermore, there is evidence in the literature that psychosocial stress at work may be a risk factor for the consumption of psychotropic substances. One large study from northern Germany found a significant association between psychosocial stress at the workplace and nicotine dependence [7]. Another prospective study from Britain [8] as well as a cross-sectional study from Russia, Poland and the Czech Republic [9] found an association between psychosocial stress and problematic alcohol consumption.

In addition, there is literary evidence that psychosocial stress at work may be a risk factor for a poor psychological state. The results of the prospective Gazel cohort study strongly support the possibility that psychosocial factors at work are predictive of depressive symptoms [10,11]. A German study on 430 medical doctors found an association between the consumption of substances and a poor psychological state [12]. Disturbances in the psychological state, like depressive symptoms, were also observed in veterinarians in the study from New Zealand [4].

It may also be taken into consideration, that veterinarians have access to prescription drugs. One case report published by Lundberg [13] describes the history of a German veterinary surgeon, which started to use intravenous morphine to reduce stress and got addicted for seven years. However, the association between stress and the consumption of psychotropic substances has not yet been examined for veterinarians.

The present study on veterinarians investigates associations between psychosocial stress, demoralization and the consumption of tobacco, alcohol and medical drugs.

## Materials and methods

### Study population

In 2006 (April/May), a self-administered questionnaire was sent to 2,012 veterinarians in Hamburg, Bremen, Schleswig-Holstein and Mecklenburg-Western Pomerania. 1,136 veterinarians participated. Complete information was provided by 1,060 subjects (response rate 52.7%).

By using the registration data from the Federal Veterinarian Council [14], responders could be compared with all registered veterinarians regarding age and sex. The proportion of women in the present study (52.9%) was higher than in the whole population of German veterinarians (46.8%). No statistically significant difference was found for sex or age. Therefore the sample can be accepted as representative.

### Measures

Demographic information was collected including data on gender, age, type of work, weekly working hours and the number of years of professional work.

Psychosocial stress during the previous 30 days was determined with a self-developed score. There are valid and reliable models and instruments for the evaluation of psychosocial stress in the literature, e.g. the job demand – control Model [15] or Siegrist's model of effort-reward imbalance at work (ERI) [16]. But no suitable instrument was found for the audience of our study, because these models are developed to evaluate psychosocial stress in general irrespective of the mode of work or the profession. The focus of our study was to determine special work demands and occupational stressors in the work field of veterinarians in Germany. Furthermore established instruments refer to employees, but veterinarians are often self-employed.

A literature search was used to identify situations of the normal job routine and to identify studies and questionnaires with an focus on stress in the veterinary profession [1-3,5,6]. With this background, 15 Items have been established. Three items were adapted from the German version of the ERI questionnaire and two items were adapted from another study on veterinarians [6] using a slightly changed format. The remaining items have been developed on the basis of the results of a two-day-coaching to reduce stress and to increase traffic safety for veterinarians, initiated by the Institution for Statutory Accident Insurance and Prevention in the Health and Welfare Services [17]. To test the items expert interviews with three veterinarians have performed. The final version of the scale consisted of the following items:

• „I am under constant time pressure due to a heavy workload.“ (ERI1)

• „I am often pressured to work overtime.“ (ERI4)

• „Over the past few years, my job has become more and more demanding.“ (ERI6)

• „During my work I am afraid of injuries and infections.“

• „Sometimes I have to deal with difficult customers.“

• „It happens that my customers call at night.“

• “I guarantee my customers that I am available 24 hours a day.“

• „The cooperation with my colleagues is sometimes difficult.“ [6]

• „There is a strong competition in my job routine.“

• „At the weekends I have to work in the emergency veterinary service.“

• „It is hard to find a regulation for holidays which corresponds to my needs.“

• „I have difficulties in balancing my professional life and private life.“ [6]

• „I think I don't have sufficient free time.“

• „I worry about my professional future.“

• “My professional achievements do not gain enough recognition.”

The scale to measure psychosocial stress refers to the stress-strain concept of Rohmert and Rutenfranz [18,19]. Within this concept, stress or stressors are defined as external factors, which have an psychogenic effect and strain is defined as the individual consequences of these external factors in a person, depending on the individual condition of the person.

Therefore the questions are answered in two steps. First, subjects disagree or agree whether or not the item describes a typical experience of their work situation. Secondly, subjects who agree are asked to evaluate to what extent they feel distressed. Each item could be answered using one of 5 replies reaching from 0 = "Disagree, and I am not at all distressed" to 4 = "Agree, and I am very distressed". The scale was tested in a pretest. The items gave a Cronbach's α = 0.86, indicating that they are reliable. The score for psychosocial stress was calculated by adding the points for each item. The higher the score values were, the more perceived demands are experienced as stressful. The observed range of psychosocial stress was 1–53 points. Values in the upper third (37 to 53 points) of the observed range were classified as intense psychosocial stress, values from 19–36 as intermediate stress and values from 0–8 as low.

A short form with 7 items of the Psychiatric Epidemiology Research Interview (PERI) Demoralization Scale was used to determine the psychological state [20,21]. Demoralization describes a non-specific indicator for disturbances in the psychological state and is accompanied by feelings such as despondency, discouragement and a negative self-assessment [20,21]. The 7 items in the scale record the occurrence of the symptoms of demoralization during the previous 30 days; possible answers range from 0 = "almost never" to 4 = "almost always". The direction of the rating had to be inverted for two items (0 = "almost always", 4 = "almost never"). The 7 items yielded a Cronbach's α = 0.81, indicating that they are reliable. A sum score was constructed by adding the points for each item; the observed range was 0–24 points. The higher the score values were, the more the veterinarians were suffering from demoralization. For the statistical analysis, we collapsed the sum score into 3 levels: high demoralization values (17 to 24 points), intermediate demoralization values (9–16 points) and low demoralization (0–8 points).

In accordance with the definition of the World Health Organization, persons who had consumed fewer than 100 items of tobacco goods during their lives were defined as non-smokers [22]. Former smokers are persons who have consumed more than 100 items during their lives, but who have abstained during the previous 30 days. Smokers are persons who have consumed tobacco goods during the previous 30 days. The average number of tobacco items smoked per day by smokers was recorded with a frequency-quantity index. A similar index has been used in other studies and has proved to be valid [12,22].

The average amount of pure alcohol in gram per day consumed during the previous 30 days was recorded with a drink-specific (beer, wine or sparkling wine, spirits) frequency-quantity index [23,24]. On the basis of the threshold values of the German Society for nutrition, the thresholds for high-risk alcohol consumption were taken as > 20 g pure alcohol per day for men and > 10 g pure alcohol for women [25].

Information on binge drinking was recorded separately by asking for the frequency of consumption of five or more glasses of alcoholic drinks on a single occasion during the previous 30 days [23]. Regular binge drinking was defined as binge drinking at least once per week.

The alcohol screening test CAGE according to Ewing [26] was used in the study (possible scores 0 to 4). The CAGE questions are: (1) "Have you ever felt you should cut down on your drinking?" (2) "Have people annoyed you by criticizing your drinking?" (3) "Have you ever felt bad or guilty about your drinking?" (4) "Have you ever had a drink first thing in the morning to steady your nerves or to get rid of a hangover?" If two or more of these questions are answered with "yes", this is an indicator for alcoholism or problem drinking (score ≥ 2). Because of its shortness and easy handling, the CAGE test is very suitable for a self-administered questionnaire and has been described as a valid instrument in other studies [27,28].

Drug intake during the previous 30 days was recorded separately for tranquilizers or sedatives, appetite suppressants or stimulants, analgesics and neuroleptics. These groups of drugs are often mentioned in the context of abuse and dependency or misuse [29]. Finally, it was asked whether the drug intake was medically necessary and prescribed for a chronic disease. Regular intake is defined as use of medical drugs at least once per week.

20 veterinarians were selected for the pretest; they were excluded from the later data analysis. As one veterinarian changed address, the sample size was reduced to 19. 10 subjects (52.6%) participated in the pretest. Only minor changes to the questionnaire had to be made after the pretest.

### Statistical analysis

The data were described with univariate and descriptive analytical methods. Multivariate analysis was calculated with multiple logistic regression models for the following dependent variables (all variables had been dichotomized):

• Intense psychosocial stress

• High demoralization values

• Tobacco consumption ≥ 10 items per day

• High-risk alcohol consumption

• Binge drinking at least once a week

• Problem drinking (CAGE score ≥ 2)

• Regular drug use

Model construction was performed stepwise. Variables with p > 0.1 were excluded if they failed to change the odds ratio (OR) of other variables [25]. The level of significance was set at p < 0.05. The 95% confidence interval (95% CI) is given. In general the unexposed group (low score) was used as reference group. For the variable "professional work", the category "non-clinical area of work" was selected as reference group, to allow for a comparison between practicing veterinarians and other veterinarians. For the variable "working hours", the category "21–40 h per week" was defined as reference, as the average number of weekly working hours for people in full employment in Germany is 39.9 h [30]. All analyses were carried out using SPSS Version 11.5.1. The study protocol was approved by the ethics committee of the Hamburg Medical Council.

## Results

The study population is described in Table 1. Most of the veterinarians (39.6%) were between 35 and 44 years old. About half (49.9%) of the study participants were practice owners, 22.5% were employed in a practice and 27.5 worked in a non-clinical area (Department of Veterinary Services, Animal Feed, Nutrition or Pharmaceutical Industry, Official Ante- and Post-mortem Meat Inspection, University). The average working hours were 47.9 h per week and 14.5% of the subjects worked more than 60 h per week.

**Table 1:** Central Study Variables (n = 1060)

		**N**	**%**
**Gender**	Men	499	47.1
	Women	561	52.9

**Age**	≤ 34 years	175	16.5
	35 – 44 years	420	39.6
	45 – 54 years	259	24.4
	≥ 55 years	206	19.5

**Professional work**	Non-clinical area of work^1^	292	27.5
	Practice owner	529	49.9
	Employee in a practice	239	22.6

**Working Hours**	≤ 20 h per week	72	6.8
	21–40 h per week	261	24.6
	41–60 h per week	573	54.1
	≥ 60 h per week	154	14.5

**Psychosocial Stress**	Low (points 1–18)	499	47.1
	Intermediate (19–36 points)	473	44.6
	Intense (37–53 points)	88	8.3

**Demoralization**	Low (0–8 points)	611	57.6
	Intermediate (9–16 points)	388	36.6
	High (17–34 points)	61	5.8

**Tobacco Consumption**	No tobacco consumption	857	80.8
	Tobacco Consumption: 1–9 items per day	110	10.4
	Tobacco Consumption: ≥ 10 items per day	93	8.8

**Alcohol Consumption**	No alcohol consumption	135	12.7
	Low-risk alcohol consumption	587	55.4
	High-risk alcohol consumption	338	31.9

**Binge drinking (≥ 5 intakes in one drinking situation)**	On no day	828	78.1
	1 to 3 times during the previous 30 days	459	15.0
	Regular (at least weekly)	73	6.9

**CAGE Test**	Score < 2	922	87.0
	Score ≥ 2 (problem drinking)	138	13.0

**Drug Use**	No intake	452	42.6
	1 to 3 times during the previous 30 days	398	37.5
	Regular intake (at least weekly)	210	19.8

**Drugs Prescribed by Doctor**	1 to 3 times during the previous 30 days	12	1.1
	Regular intake (at least weekly)	41	3.9

^1^Department of Veterinary Services, Animal Feed, Nutrition or Pharmaceutical Industry, Official Ante- and Post-mortem Meat Inspection, University

### Psychosocial stress and demoralization

For psychosocial stress the respondents rated the items "time pressure due to a heavy workload" (26.7%), "difficulties in balancing professional life and private life" (24.1%), "dealing with difficult customers" (22.5%) and "insufficient free time" (22.4%) as highly or very highly stressful (no table). Overall 8.3% of subjects reported intense psychosocial stress (Table 1). Veterinarians in the intermediate age group more often reported psychosocial stress than their colleagues aged over 54 (35 to 44 years: OR 2.3, 95% CI 1.0–5.0; 45 to 54 years: OR 3.2, 95% CI 1.4–7.2). Practice owners (OR 5.8, 95% CI 2.4–22.9) and veterinarians employed in a practice (OR 7.4, 95% CI 2.0–16.7) are more frequently affected than veterinarians working elsewhere. The probability of intense psychosocial stress increased with the number of working hours (41–60 h: OR 5.2, 95% CI 1.8–14.6; > 60 h: OR 16.4, 95% CI 5.7–47.8) (Table 2).

**Table 2:** Adjusted Odds Ratios for Intense Psychosocial Stress and for High Demoralization Values

**Intense Psychosocial Stress Variables in the Model**		**%**	**Crude OR (95% CI)**	Adjusted OR* (95% CI)
Age	≤ 34 years	8.6	2.1 (0.8–4.8)	1.7 (0.6–4.8)
	35 – 44 years	8.6	2.1 (0.9–4.3)	2.3 (1.0–5.0)
	45 – 54 years	10.8	2.7 (1,2–5.8)	3.2 (1.4–7.2)
	≥ 55 years	4.4	1	1

Professional Work	Non-clinical area of work^1^	1.4	1	1
	Practice owner	11.2	9.0 (3.2–25.1)	5.8 (2.0–16.7)
	Employee in practice	10.5	8.4 (2.9–24.5)	7.4 (2.4–22.9)

Working Hours	≤ 20 h per week	4.2	2.8 (0.6–12.8)	2.4 (0.5–10.9)
	21–40 h per week	1.5	1	1
	41–60 h per week	7.5	5.2 (1.9–14.7)	5.2 (1.8–14.6)
	≥ 60 h per week	24.7	21.0 (7.3–60.3)	16.4 (5.7–47.8)

**High Demoralization Values Variables in the Model**		**%**	**Crude OR (95% CI)**	Adjusted OR* (95% CI)

Working Hours	≤ 20 h per week	11.1	2.2 (0.8–5.5)	1.8 (0.7–4.7)
	21–40 h per week	5.4	1	1
	41–60 h per week	5.2	1.0 (0,5–1.9)	0.5 (0.2–1.0)
	≥ 60 h per week	5.8	1.1 (0.5–2.6)	0.3 (0.1–0.7)

Psychosocial Stress	Low (1–18 points)	2.4	1	1
	Intermediate (19–36 points)	5.7	2.5 (1.2–4.9)	3.3 (1.6–6.8)
	Intense (37–53 points)	25.0	13.5 (6.4–28.6)	24.8 (10.5–58.4)

* Gender and Working Years had no effect.

** Gender, Age, Professional Work, Working Years had no effect.

^1^Department of Veterinary Services, Animal Feed, Nutrition or Pharmaceutical Industry, Official Ante- and Post-mortem Meat Inspection, University

On the Demoralization Scale 17.1% of respondents rated that they are almost always/frequently dissatisfied with themselves; 16.7% rated that they are almost never/rarely optimistic and confident and 15.6% almost never/rarely feel proud (no table). Overall 5.8% of subjects gave high demoralization values (Table 1).

Veterinarians who work fewer than 21 h per week are almost twice as frequently affected by demoralization (11.1%) than others. However, this difference is not statistically significant. Working more than 60 h per week has a protective effect on demoralization (OR 0.3, 95% CI 0.1–0.7). The probability of high values for demoralization increases with increasing psychosocial stress (Table 2).

### Tobacco consumption

More than half (55.3%) of veterinarians are non-smokers; 25.5% are former smokers; 19.2% are smokers.

8.8% consumed ≥ 10 items of tobacco goods per day (Table 1). This high level of consumption was found about twice as often in men than in women (OR 2.1; 95% CI 1.4–3.3). Subjects with high demoralization values more often consumed ≥ 10 items per day (OR 2.8, 95% CI 1.3–6.0). Age, professional work, working hours and psychosocial stress had no effect on tobacco consumption (no table).

### Consumption of alcoholic drinks

4.1% of veterinarians had been abstinent for at least a year; 8.6% had consumed no alcoholic drink within the previous 30 days and 87.3% had consumed alcohol within this period. The consumers included 90.8% of men and 84.1% of women.

Most of the veterinarians consumed various forms of alcoholic drinks (beer, wine or spirits) during the previous 30 days. Men consumed a mean of 15.9 g (± 17.1 g) pure alcohol per day and women 9.2 g (± 12.3 g) per day (no table).

#### High-risk Alcohol Consumption

31.9% of the subjects practiced high-risk consumption (Table 1). Women practiced high-risk consumption more frequently than men (OR 1.3, 95% CI 1.0–1.8). High-risk alcohol consumption was found more often in practice owners than in veterinarians employed in a practice or working elsewhere (OR 1.4, 95% CI 1.2–2.1) (Table 3).

**Table 3:** Adjusted Odds Ratios for the Outcome Measures for the Consumption of Alcoholic Drinks

**High-risk Alcohol Consumption Variables in the Model**		**%**	**Crude OR (95% CI)**	Adjusted OR* (95% CI)
Gender	Male	30.3	1	1
	Female	33.3	1.2 (0.9–1.5)	1.3 (1.0–1.7)

Professional work	Non-clinical area of work^1^	29.1	1	1
	Practice Owner	36.5	1.4 (1.0–1.9)	1.4 (1.1–2.0)
	Employee in a Practice	25.1	0.8 (0.6–1.2)	0.8 (0.5–1.1)

**Regular Binge Drinking Variables in the Model**		**%**	**Crude OR (95% CI)**	Adjusted OR** (95% CI)

Gender	Male	11.8	5.2 (2.9–9.5)	5.1 (2.8–9.3)
	Female	2.5	1	1

Psychosocial Stress	Low (1–18 points)	6.0	1	1
	Intermediate (19–36 points)	6.6	1.1 (0.7–1.8)	1.0 (0.6–1.8)
	Intense (37–53 points)	13.6	2.5 (1.2–5.0)	2.2 (1.1–4.5)

**Problem Drinking (CAGE ≥ 2) Variables in the Model**		**%**	**Crude OR (95% CI)**	Adjusted OR*** (95% CI)

Professional Work	Non-clinical area of work^1^	11.3	1	1
	Practice owner	17.0	1.6 (1.1–2.5)	1.7 (1.1–2.5)
	Employee in a practice	6.3	0.5 (0.3–1.0)	0.5 (0.3–0.9)

Demoralization	Low (0–8 points)	10.5	1	1
	Intermediate (9–16 points)	14.9	1.5 (1.0–2.2)	1.6 (1.1–2.4)
	High (17–34 points)	26.2	3.0 (1.6–5.7)	3.4 (1.8–6.5)

* Age, working hours, psychosocial stress and demoralization had no effect.

** Age, professional work, working hours, working years and demoralization had no effect.

*** Gender, age, working hours, working years and psychosocial stress had no effect.

^1^Department of Veterinary Services, Animal Feed, Nutrition or Pharmaceutical Industry, Official Ante-and Post-mortem Meat Inspection, University.

#### Binge Drinking

21.9% of the veterinarians reported binge drinking on at least one occasion during the previous 30 days. 6.9% reported regular binge drinking, i.e. at least once a week (Table 1).

Binge drinking was found more often in men than in women (OR 5.1, 95% CI 2.8–9.3). Veterinarians under intense psychosocial stress are about twice as often affected than their colleagues (OR 2.2, 95% CI 1.1–4.5) (Table 3).

#### The CAGE Alcohol Screening Test

Overall 13.0% of the veterinarians screened positive for problem drinking according to CAGE (Score ≥ 2) (Table 1). The rate of positive screening results among men was 15.2% and among women 11.1%. However, this difference is not statistically significant. Men who screened positive according to CAGE consume a mean of 33.6 g (± 13.0 g) pure alcohol per day and women consume a mean of 24.6 g (± 19.0 g) per day (no table).

Veterinarians who are practice owners are more often affected than veterinarians with other occupations (OR 1.7, 95% CI 1.1–2.5). On the contrary, veterinarians employed in a practice are more rarely identified as problem drinkers (OR 0.5, 95% CI 0.3–0.9). For veterinarians with intermediate values for demoralization, the OR was 1.6 (95% CI 1.1–2.4). For high demoralization values, the OR increased to 3.4 (95% CI 1.8–6.5) (Table 3).

### Medical Drug use

57.4% of the veterinarians had taken a drug from one of the relevant groups within the preceding 30 days. 5.0% (n = 53) of the drugs had been medically prescribed by a doctor. About one in five (19.8%) used one of the drugs regularly, i.e. at least once a week (Table 1).

• 18.3% of the veterinarians regularly used analgesics. 4.2% had a prescription for these and one of this persons used an opioid (Tramadolor).

• 1.3% of the veterinarians regularly used sedatives or tranquilizers. With a single exception, this was in the form of self-medication.

• 1.6% of the veterinarians regularly used antidepressives. 0.7% of these drugs had been prescribed.

• 0.4% (n = 4) of the veterinarians took appetite suppressants or stimulants without a medical prescription. Three of these four individuals are women. However, these preparations were not consumed regularly.

• 0.2% (n = 2) of the veterinarians took neuroleptics. One of these individuals did this regularly on medical prescription

• In summary 2.4% (n = 25) had used drugs with a psychotropic effect (tranquilizer, antidepressives, opioid, neuroleptics) within the preceding 30 days and 2.0% (n = 21) did this regularly (no table).

Table 4 shows the results for regular medical drug intake. Women (OR 1.4, 95% CI 1.0–2.0) and employees in practices (OR 1.6, 95% CI 1.0–2.6) more regularly took drugs. Intense psychosocial stress (OR 1.9, 95% CI 1.1–2.8) and intermediate (OR 2.0, 95% CI 1.4–2.8) and high demoralization values (OR 2.9, 95% CI 1.6–5.3) are risk factors for regular drug use.

**Table 4:** Adjusted Odds Ratios for Regular Drug Use

**Regular Use Variables in the Model**		**%**	**Crude OR (95% CI)**	Adjusted OR* (95% CI)
Gender	Male	16.0	1	1
	Female	23.2	1.6 (1.2–2.2)	1.4 (1.0–2.0)

Professional Work	Non-clinical area of work^1^	15.4	1	1
	Practice owner	18.5	1.2 (0.8–1.8)	1.2 (0.8–1.7)
	Employee in a practice	28.0	2.1 (1.4–3.3)	1.6 (1.0–2.6)

Psychosocial Stress	Low (1–18 points)	14.4	1	1
	Intermediate (19–36 points)	22.8	1.8 (1.3–2.4)	1.4 (0.9–2.0)
	Intense (37–53 points)	34.1	3.1 (1.8–5.1)	1.9 (1.1–3.4)

Demoralization	Low (0–8 points)	13.4	1	1
	Intermediate (9–16 points)	27.1	2.4 (1.7–3.3)	2.0 (1.4–2.8)
	High (17–34 points)	37.7	3.9 (2.2–6.9)	2.9 (1.6–5.3)

* Age, working hours, working years had no effect.

^1^Department of Veterinary Services, Animal Feed, Nutrition or Pharmaceutical Industry, Official Ante-and Post-mortem Meat Inspection, University.

## Discussion

Our study is the first approach to investigate the association of psychosocial stress, demoralization and the consumption of psychotropic substances in the veterinary profession. Due to the limited knowledge in this field we conducted a cross-sectional study using self-administered questionnaires. We obtained a high response rate of about 53% and we could find no differences between the responders and the non-responders in sex or age. Nevertheless there might be some bias due to the selection of non-responders.

Self-reported data on the consumption of alcohol, tobacco and medical drugs may be biased in the direction of what is socially desirable. For this reason, questions and instruments like the CAGE test were used which had already been proven in other studies on substance use.

To our knowledge the Demoralization Scale was used for the first time to examine the association of disturbances in the psychological state and the use of psychotropic substances.

We developed a job-specific score for psychosocial stress, which has not been formally validated. This may limit the generalizability of our results.

As changes with time cannot be considered in a cross-sectional study, the OR can provide evidence for factors influencing the results, but only restricted conclusions about the causality of these correlations are possible. It would therefore be desirable to confirm these findings by additional research.

### Psychosocial stress and demoralization

Time pressure due to a heavy workload, difficulties in balancing one's professional and personal life, dealing with difficult customers and insufficient free time were regarded as causes of psychosocial stress. Veterinarians working in practices were exposed to an increased risk. More than two-thirds of the veterinarians in Germany are working in a practice [14]. During their typical work-life, veterinarians in practices deal with on-call duties and/or emergency veterinary service at the weekends. On the other hand most of the veterinarians are self-employed as the practice owner and they have to acquire their customers to compete with other practices. In addition, veterinarians in large animal practices have to deal with client home visits to treat the animals and with customers (e.g. farmers, rancher) under high economic pressure.

The probability of psychosocial stress increased with the number of hours worked per week. Veterinarians work a mean of 47.9 h per week, which is much higher than the mean figure of 39.9 h per week for fully employed persons in Germany [30].

Other studies have come to similar results. As mentioned above Trimpop et al. [6] conducted a study on accidents and stress in German veterinary practices. 778 veterinarians participated in the study and it was shown that the mean number of hours worked per week was 51.5 h and that there was a correlation between the high number of working hours and stress. Gardner and Hini [4] performed a study on work-related stress in veterinarians in New Zealand, in which 927 veterinarians took part (response 48.6%). They concluded that veterinarians suffer stress because of the number of working hours, the expectations of their customers and from unexpected events. In a longitudinal Australian study reported by Heath [5], 124 veterinarians with 10 working years after completing university were asked about their attitudes to their profession. More than two thirds reported that they suffered considerable stress from work and that they had difficulty in combining their professional and personal lives.

About 6% of veterinarians reached high values of demoralization. In other words, they often suffer from symptoms of demoralization. Demoralized veterinarians report that they are often dissatisfied with themselves. According to Gardner und Hini [4], the expectation of themselves was a factor which caused stress in veterinarians.

Risk factors for demoralization include moderate and, especially, intense psychosocial stress. This is in accordance with the hypothesis that psychosocial stress can trigger disturbances in the psychological state. Demoralized veterinarians often work ≤ 20 h per week. On the other hand, a working time of ≥ 60 h per week has a protective effect with respect to demoralization, after correction for psychosocial stress. It should therefore be assumed that there must be a complex interrelationship between working hours and demoralization which could not be clearly mapped because of the cross-sectional design. For example, demoralization may be caused by the lack of professional demands, if lack of work leads to low working hours. Otherwise, it could be assumed that demoralized people are not able to work more and that they therefore reduced their working schedule or do not remain in the profession. Other studies have not covered the relationship between stress, working hours and demoralization.

### Tobacco consumption

19.4% of veterinarians are smokers. The 2003 Telephone Health Survey, based on a randomly generated sample of 8318 persons in the resident German-speaking population (response rate 59.2%) measured the much higher prevalence of 32.5% for smokers [31].

Veterinarians belong to the upper social class and the 1998 National Health Survey found that there are fewer smokers in the social upper class (27.6%) than in the social lower class (36.8%) or middle class (32.9%). The 1988 National Health Survey was performed using a written questionnaire and a medical investigation on 7124 subjects; their social class was recorded using the Winkler and Stolzenberg multidimensional index [32].

The 1995 microcensus found the lowest prevalence of smoking in professional groups (teachers and doctors) who were comparable to veterinarians with respect to their educational standard. The microcensus is an official representational statistic; 0.5% of the resident population (95359 persons) were interviewed in 1995 on questions including their smoking habits [33].

Men and demoralized individuals exhibit an increased risk of consuming ≥ 10 items of tobacco goods per day. Other studies have also concluded that men are more often smokers and that they consume more cigarettes per day than women [22,31,32,34]. The other studies did not investigate any connection between demoralization and tobacco consumption.

### Alcohol consumption

90.5% of the men and 84.5% of the women had consumed alcohol during the preceding 30 days. In the 2003 Epidemiological Survey on Substance Abuse, 8061 persons (response 55%) in the resident German population were asked about their consumption of illegal drugs, alcohol, tobacco and narcotics. It was found that 86.8% of the men and 79.1% of the women drank alcohol during this period [23]. According to this, veterinarians drink alcohol more often than the rest of the population.

### High-risk alcohol consumption, regular binge drinking and the CAGE test

In contrast to other studies, it was striking that the female veterinarians more often reported high-risk consumption than their male colleagues (33.3% versus 30.3%). However, the 1988 National Health Survey found that women of high socioeconomic status (30%) more often practice high-risk alcohol consumption than women of intermediate (14%) or lower status (9%). The influence of socioeconomic status is apparently less marked for the male consumers (higher status: 35%, intermediate status: 29%, lower status: 32%) [25].

According to Lademann and Kolip, high alcohol consumption may be traditionally more a male role, but the recent behaviour of women of higher socioeconomic status may indicate that this level of alcohol consumption is also compatible with the female role [34].

6.9% of veterinarians engage regularly in binge drinking. These were mostly men. In the 2003 Epidemiological Survey on Substance Abuse, about twice as many persons (12.9%) regularly practiced binge drinking, including a majority of men [23].

The risk of regular binge drinking in veterinarians is markedly increased for veterinarians under intense psychosocial stress. Psychosocial factors at the workplace and problematical alcohol consumption by men was also investigated by a cross-sectional study using the data of the pilot HAPIEE Study (Health, Alcohol and Psychosocial factors In Eastern Europe). Data were analysed from 694 men from Russia, Poland and the Czech Republic. It was found that there was a association between effort-reward imbalance at work and increased alcohol consumption [9]. Most recently, a cross-sectional study by Frone [35] based on a sample of 2790 workers from the National Survey of Workplace Health and Safety (U.S.) (response rate 57%), explored the relations of 2 work stressors (work overload and job insecurity) to employee alcohol use. The results fail to support a relation between work stressors and the overall measures of alcohol use, but the results support a relation between work stressors and alcohol use during the workday and after work [35].

According to the CAGE test, the alcohol consumption of 13.0% of veterinarians is problematical. Problem drinkers – both men and women – exhibit a mean alcohol intake in excess of the threshold for high-risk alcohol consumption. In addition, demoralization is a risk factor for problematical consumption according to CAGE.

In a study on substance use by doctors during practical training, in which 431 persons (response rate 51%) provided information, the alcohol consumption of 13% was problematical according to the CAGE test. This group also more often reported that their psychological state was poor or moderate [12].

In contrast, the prevalence of problematical consumption according to CAGE in the general population is lower. Kraus et al. [24] performed a study on alcohol use and the association between onset of use and alcohol-related problems in a general population sample in Germany. In this study 7501 persons were asked about their alcohol consumption. This was based on telephone interviews between 1994 and 1996, using the CAGE test. According to the CAGE test, 8.1% of the population were problem drinkers.

A prospective British study investigated whether psychosocial factors at the workplace influence problematical alcohol consumption. 7372 civil servants (response 72%) took part at all three times of data recording. A correlation was found for men between problematical alcohol consumption according to CAGE and effort-reward imbalance at work. There was a similar trend for women, although this was not significant [8].

### Regular medical drug use

About one in five veterinarians (19.8%) reported regular medical drug use, with analgesics being by far the most important group. Women more often take drugs regularly. Veterinarians in practice are more often affected than their colleagues in a non-clinical area of work. Most of the drugs are taken without medical prescription. The risk of drug consumption is increased for persons under psychosocial stress and for demoralized persons.

Other studies have reached similar conclusions. In the study with doctors in training, 19.0% reported regular use. Most of these were women. Analgesics were most often taken. For 93% of subjects, the analgesics were taken as self-medication [12].

In the 2003 Epidemiological Survey on Substance Abuse, 16.8% of persons regularly took drugs. Women reported more regular drug intake. Analgesics were more often taken than other drugs. In addition the Survey employed four questions which reflected a drug-orientated way of life. 6.8% of those questioned reported that they only felt "half human" without drugs; 9.6% thought they were unable to master the day without drugs; 7.3% could not do without sedatives or tranquilizers in some situations [29].

## Conclusion

Complex interrelationships between psychosocial stress, demoralization and the consumption of psychotropic substances were identified.

Psychosocial stress is associated with binge drinking, with regular medical drug consumption and with demoralization. This, again, is in relation with tobacco consumption, problem drinking and medical drug consumption. Firstly, these results indicate that a strategy to cope with psychosocial stress in the veterinary profession might be the consumption of psychotropic substances. Secondly, these results show that psychosocial stress might lead to disturbances in the psychological state in the form of demoralization. And thirdly, demoralization itself may lead to an increased consumption of psychotropic substances. That means that it would be important to reduce psychosocial stress in the veterinary profession, in order to reduce the consumption of psychotropic substances and demoralization. Therefore it is important to know which factors are associated with psychosocial stress. The main factors found in our study for psychosocial stress were time pressure due to heavy workload, difficulties in balancing one's professional and personal life, dealing with difficult customers, insufficient free time and long working hours. These factors should be considered if programs or strategies to reduce psychosocial stress in the veterinary profession are implemented. Moreover, there were also differences with respect to the professional work. Practicing veterinarians, especially veterinarians owning a practice, are more frequently affected by psychosocial stress and have a greater risk of drug or alcohol consumption. These differences should also be considered.

However, due to the cross-sectional design of our study the direction of the associations found is not clear, but the findings underscore the need for examining the processes linking psychosocial stress, demoralization and the use of psychotropic substances by veterinarians. Further research, e.g. in form of a longitudinal study, is needed to confirm the causality of our findings.

## Competing interests

The authors declare that they have no competing interests.

## Authors' contributions

PS has made substantial contributions to conception and design of the study. She has been involved in revising the manuscript critically for important intellectual content. She has given final approval of the version to be published. AS has made substantial contributions to interpretation of data. She has been involved in revising the manuscript critically for important intellectual content. She has given final approval of the version to be published. AN has made substantial contributions to conception and design, as well as to analysis and interpretation of data. He has been involved in drafting the manuscript. He has given final approval of the version to be published. MH has made substantial contributions to conception and design, acquisition of data, as well as to analysis and interpretation of data. She has been involved in drafting the manuscript. She has given final approval of the version to be published.
